# Disrupted Nitric Oxide Metabolism from Type II Diabetes and Acute Exposure to Particulate Air Pollution

**DOI:** 10.1371/journal.pone.0144250

**Published:** 2015-12-14

**Authors:** Ashley P. Pettit, Howard Kipen, Robert Laumbach, Pamela Ohman-Strickland, Kathleen Kelly-McNeill, Clarimel Cepeda, Zhi-Hua Fan, Louis Amorosa, Sara Lubitz, Stephen Schneider, Andrew Gow

**Affiliations:** 1 Graduate School of Biomedical Sciences, Robert Wood Johnson Medical School, Rutgers University, Piscataway, NJ, 08854, United States of America; 2 Robert Wood Johnson Medical School, Rutgers University, Piscataway, NJ, 08854, United States of America; 3 Environmental and Occupational Health Sciences Institute, Piscataway, NJ,08854, United States of America; 4 School of Public Health, Rutgers University, Piscataway, NJ,08854, United States of America; 5 Robert Wood Johnson University Hospital, New Brunswick, NJ, 08901, United States of America; 6 Ernest Mario School of Pharmacy, Rutgers University, Piscataway, NJ, 08854, United States of America; University of Edinburgh, UNITED KINGDOM

## Abstract

Type II diabetes is an established cause of vascular impairment. Particulate air pollution is known to exacerbate cardiovascular and respiratory conditions, particularly in susceptible populations. This study set out to determine the impact of exposure to traffic pollution, with and without particle filtration, on vascular endothelial function in Type II diabetes. Endothelial production of nitric oxide (NO) has previously been linked to vascular health. Reactive hyperemia induces a significant increase in plasma nitrite, the proximal metabolite of NO, in healthy subjects, while diabetics have a lower and more variable level of response. Twenty type II diabetics and 20 controls (ages 46–70 years) were taken on a 1.5hr roadway traffic air pollution exposure as passengers. We analyzed plasma nitrite, as a measure of vascular function, using forearm ischemia to elicit a reactive hyperemic response before and after exposure to one ride with and one without filtration of the particle components of pollution. Control subjects displayed a significant increase in plasma nitrite levels during reactive hyperemia. This response was no longer present following exposure to traffic air pollution, but did not vary with whether or not the particle phase was filtered out. Diabetics did not display an increase in nitrite levels following reactive hyperemia. This response was not altered following pollution exposure. These data suggest that components of acute traffic pollution exposure diminish vascular reactivity in non-diabetic individuals. It also confirms that type II diabetics have a preexisting diminished ability to appropriately respond to a vascular challenge, and that traffic pollution exposure does not cause a further measureable acute change in plasma nitrite levels in Type II diabetics.

## Introduction

Epidemiological and toxicological studies have amply demonstrated associations between acute and chronic exposure to traffic-related air pollutants (TRAPs) and cardiorespiratory health effects [1]. TRAPs are a dynamic mixture of particles and gaseous compounds that arise from multiple sources. For many individuals exposure to substantial amounts of air pollutants likely occurs during daily commuting or at home when individuals live in close proximity to roads with high traffic volume [2–5]. A case- crossover study found that short term exposures to traffic pollution increased the risk of non-fatal myocardial infarction by 2 fold within a few hours of exposure [6]. Generally, mechanisms for health effects resulting from relatively brief exposures to TRAPs are not well understood. Diesel exhaust, an important contributor to TRAPs, has been found in human studies to cause inflammatory responses, adverse changes in cardiovascular biomarkers, and reversible health effects in response to controlled, acute exposures [7–12]. A small number of studies have investigated real world, acute exposures to TRAPs near roadways. These studies have shown effects on heart rate, respiratory inflammatory responses, adverse effects on lung function and alterations in oxidative stress [13–17].

The relative importance of the various components of the complex TRAPs mixture, as well as the biological pathways by which different TRAPs components may cause adverse health effects, are not well-understood. Exposure to TRAPs, diesel exhaust and particles increases inflammatory responses in animal and human respiratory tracts, in addition to cell systems. Oxidative stress is considered to be a key mechanism in these adverse responses [7, 17–22].

In previous studies, we found that nitrite and nitrate, relatively stable oxidation products of nitric oxide metabolism, were increased in exhaled breath condensate (EBC) among asthmatics after controlled exposure to diesel exhaust [9] and sub acutely following exposures to high levels of ambient pollutants, predominantly from traffic [17]. More recently, we demonstrated a similar effect in exhaled breath condensate immediately following a 1.5-hour highway exposure to TRAP [23]. The effects of TRAPs exposure on vascular oxidative stress have been established, but its impact on the vascular endothelium has yet to be examined.

Type 2 diabetes mellitus (T2DM) is associated with increased risk for acute and chronic cardiovascular disease [24–28]. Studies suggest that a major underlying factor in these pathologies is vascular endothelial dysfunction, recognized as an independent risk factor for cardiovascular events [29, 30].

The metabolically active vascular endothelium regulates blood flow through modulation of vascular smooth muscle tone via production of and response to molecules such as nitric oxide (NO) [31–33]. NO mediates numerous processes including vasodilation, vascular smooth muscle proliferation, and vascular inflammation. Reduced production and bioavailability of NO is a hallmark of endothelial dysfunction [24, 25, 34–36].

An increase in tissue metabolic demands due to physiological or chemical stressors, particularly oxidative stress, normally results in hypoxic vasodilation mediated by NO. NO has a short half-life and is rapidly oxidized into nitrite (NO_2_^-^) and subsequently into nitrate (NO_3_^-^) in the vasculature. Nitrite, although shorter-lived, is considered a more meaningful reflection of NO production, as it reflects NO produced by the endothelial cell lining of blood vessels, while nitrates in circulation may originate from alternate sources such as food constituents and preservatives [37].

The ability of the vasculature to respond appropriately to stress can be measured through the response to a brief local ischemic challenge known as reactive hyperemia. Typically termed brachial artery flow-mediated dilation (BAFMD) when ultrasound measurements of arterial diameter are made, other investigators have directly measured NO metabolites in blood as a relevant endpoint [24, 37]. In healthy vascular tissue the response to occlusion is characterized by a rapid increase in both vessel size and blood flow mediated by metabolic alterations including a rapid increase in production of NO [24, 38]. Diabetics and those with cardiovascular disease (CVD) typically show a decreased ability to appropriately respond to ischemic and other stressors, termed endothelial dysfunction [39].

Previous studies have shown that adults with T2DM have reduced flow-mediated dilation [38, 40] on days with higher ambient pollution, and also have acutely decreased high frequency heart rate variability from roadway pollution [16]. Healthy adults had altered brachial artery diameter [41, 42] and endothelial function [43] after experimental exposures to concentrated ambient particulate matter (PM) or diesel exhaust.

We hypothesized that 1.5-hr rides in a passenger vehicle in morning rush-hour traffic on a major highway would cause acute increases in oxidative stress in the peripheral vasculature manifested by impaired generation of NO, observed as its metabolite nitrite, in response to a standard ischemic stimulus, and that the PM component of TRAP is responsible for these effects. We further hypothesized that the impairment in generation of NO would be greater in subjects with T2DM compared to healthy control subjects.

## Materials and Methods

### Study Population and Ethics Statement

Twenty subjects with T2DM aged 46 to 70 years and 20 non-diabetics were recruited from the community and from endocrinology clinics at RWJMS. All participants were medically cleared for the study with the following exclusions: pregnant and breast feeding women, unstable kidney/liver disease, unstable coronary artery disease or arrhythmia, peripheral arterial disease, uncontrolled hypertension, asthma, other pulmonary disease, cigarette smoking, or the inability to ride comfortably in a car for 2 hours. Prior to participation all subjects signed an informed consent approved by the UMDNJ (now Rutgers University) Internal Review Board.

### Exposure Protocol

Treating physicians adjusted diabetes medications prior to each study visit to account for the lack of breakfast on study mornings (fasting after midnight) and blood glucose was monitored before and after the ride to avoid hypoglycemia. Subjects began pre exposure testing at approximately 7am with all study testing completed within a 4-hr window. Subjects lived within 20 km of the Environmental and Occupational Health Sciences Institute (EOHSI) and were encouraged to avoid highways when driving to reach the testing facility on days of their exposures. Exposure to weekday rush-hour traffic pollution occurred in a Ford Taurus on the New Jersey Turnpike (NJTPK). Each subject rode in the rear seat of the passenger vehicle, which was driven by professional drivers. The car rides were approximately 1.5 hours in length and approximately 79 miles round trip primarily in the diesel-enriched NJTPK truck/bus lanes (6 miles on local roads, 18 miles on Interstate 287, and 55 miles on the NJTPK). Windows were maintained closed but external air intakes were open (non-recirculation mode) with the fan on medium setting. Each subject participated in two randomly ordered exposure sessions at least one week apart. A powered air purifying respirator (PAPR) was utilized for both car rides. Exposures were either filtered (PAPR with HEPA filter in the respirator housing) or unfiltered (PAPR without HEPA filter). Subjects and technicians were blinded to presence or absence of the filter, which was randomized between rides.

### Vascular Reactivity

A five minute arm occlusion consisted of inflation of a blood pressure cuff to 50mmHg above systolic blood pressure. The cuff was placed on the biceps approximately 2–3 cm superior to the olecranon process allowing access to the median cubital vein for phlebotomy.

### Blood Collection

Blood draws were taken prior to and immediately following TRAP exposure from the median cubital vein in the test arm. Samples were collected into a BD plastic whole blood tube with spray-coated K_2_EDTA and immediately placed on ice. Draws were completed at baseline and 60 seconds following release of the upper arm occlusion. Samples were fractionated by centrifugation (13000 rpm for 20 minutes). The plasma was drawn off and aliquoted into three 1.5mL plastic eppendorf tubes. One plasma aliquot was placed on ice and analyzed for nitrite within 4 hours of sampling. The remaining plasma was frozen and stored at -80°C for future biochemical analyses.

### Measurement of plasma nitrite

The NO metabolite, nitrite (NO_2_^-^), was analyzed using an ozone-based chemiluminescent technique with a Sievers Nitric Oxide Analyzer 280. NO_2_^-^ analysis was completed at room temperature utilizing potassium iodide and acetic acid as reductants and argon as an inert carrier gas. This reaction specifically reduces NO_2_^-^ into NO, but cannot reduce higher oxides of nitrogen (like nitrate (NO_3_^-^)) to NO. Standards for the assays were prepared by serial dilutions of sodium nitrite (Sigma Aldrich) with ultrapure water. Volumes of 10 μL of standards and 40 μL of sample were injected into the reaction vessel to give adequate reaction signal. Integrations of resulting peaks were calculated in the Liquid program to produce areas under the curve (AUCs) from standards and samples. Sample concentrations were calculated utilizing a formulated regression line from known standard concentrations.

### Exposure Measurements

We made the following measurements with inlets at the subject’s breathing zone inside the respirator (PAPR) face piece and separately near the center of the vehicle cabin: mass concentrations of PM with median cut-point of 2.5 μm (PM_2.5_) at 1-min intervals using TSI SidePak model AM510 aerosol monitors (TSI, Inc, Shoreview, MN) with the calibration factor set at 0.32 (based on collocated gravimetric analysis of local ambient particles); number concentrations of particles with aerodynamic diameter from 0.01 to 1.0 μm at 1-min intervals using a condensation particle counter, TSI model 3007 (TSI, Inc.). Pollutants measured in the vehicle cabin only were: back carbon using an AE-51 microaethalometer (Aethlabs, San Francisco, CA) at flow rate of 100 ml/min and 1-min time base; nitrogen dioxide (NO_2_) collected on triethanolamine-coated Sep-Pak cartridges (Waters, Corp, Millford, MA), analyzed using HPLC-UV as previously reported [44]; carbon monoxide (CO) and air temperature were measured continuously with a Langan T15v monitor (Langan, Inc, San Francisco, CA); and humidity with a HOBO 8 Pro Series monitor (Onset, Bourne, MA)

### Statistical Analysis

Demographic characteristics between diabetics and controls and pollutant concentrations within the PAPR were examined with t-tests or chi-square tests, as appropriate. Histograms were examined for normality and outliers. Geometric means and 95% confidence intervals were calculated for the raw nitrite values as well as the change due to cuff at each time point.

Linear models with random effects to account for repeated measures were used to assess the main effects of diabetic status and exposure as well as the interaction of diabetic status and exposure on the nitrite measures. Log transformations were taken of nitrite values in order to stabilize the variance and ensure that the response variables were normally distributed. When examining exposure, the pre-exposure measure was included as a covariate (accounting for regression to the mean) and a random intercept for subject controlled for correlation within subject due to repeated exposure sessions. Ratios were calculated of changes due to the filtered versus unfiltered conditions, for example, as (exp(*β*)-1)x100% where *β* was the regression coefficient associated with the dummy variable representing exposure (= 1 when unfiltered and = 0 for filtered). Confidence intervals were calculated similarly using the endpoints of the 95% confidence intervals for *β*.

## Results

### Subject Characteristics

Subject characteristics are summarized in Table 1. The control group had significantly lower systolic blood pressure (SBP), body mass index and weight than the diabetic group. Age, racial groupings, and diastolic blood pressure (DBP) did not differ between the diabetic and control groups. Diabetic subjects had Hb_A1c_ levels ranging from 7.0 to 11.2. Diabetics were prescribed a greater number of medications than controls for health issues beyond glycemic control, although 13 of the 20 controls had treated medical conditions such as hypertension or lipid abnormalities. The control group was comprised of a female majority (55%), while the diabetic cohort consisted of a male majority (75%). No subject had a blood glucose level below 90 mg/dl before or following a ride.

**Table 1:** Subject characteristics (n = 40).

	**Mean±SD**	
	**Controls**	**Diabetics**
**Age(years)**	57.4±7.0	61.0±7.9
**SBP(mmHg)** ^a^	125.7±13.5	137.5±12.9
**DBP(mmHg)**	76.1±9.6	76.7±9.2
**BMI(kg/m** ^**2**^ **)** ^a^	27.1±6.2	33.2±6.3
**Hb**_**A1c**_ **(%)**	N/A	8.6±1.3
**Weight(kg)** ^a^	76.0±18.5	93.8±19.9
	**Number of subjects**	
**Sex** **Women Men**	119	515
**Race**		
**Caucasian**	15	16
**Black**	3	3
**Other**	2	1
**Medications**		
**Diabetics medication**		
**Insulin**	0	12
**Metformin**	0	12
**Sulfonylureas**	0	12
**Dipeptidyl peptidase-4**	0	2
**Acarbose**	0	1
**Glitazone**	0	1
**Other medication**		
**Lipid lowering(statins)**	4	18
**Other(nonstatins)**	5	4
**Platelet aggregation inhibitors**	3	14
**Angiotensin-converting enzyme (ACE)inhibitors**	4	10
**Calcium channel blockers**	1	6
**Angiotensin II receptor blockers**	2	6
**Beta blockersHormone replacementMen (testosterone)Women (estrogen)**	211	610

Systolic (SBP) and diastolic blood pressures (DBP) were reported as a mean of triplicate measurements following the rest period. Body mass index (BMI) was calculated from weight and height(not reported) after measurement at the facility. Medications and hemoglobin A1c (Hb_A1c_) levels in diabetics were reported from medical records Hb_A1c_ was not measured in control subjects and medications were self-reported.

^a^p≤0.05 Controls were significantly lower when compared to diabetics.

### Exposure Characteristics

The pollutant measurements show no significant differences inside the vehicle cabin between filtered and unfiltered rides as the filter only affected the air directly inhaled by the subject through the PAPR (Table 2). However, inside the PAPR face piece mean PM_2.5_ concentration was 83.9% lower (p< 0.0001) and mean particle number concentration was 99.9% lower (p<0.0001) during HEPA-filtered rides compared to unfiltered rides.

**Table 2:** Characteristics of filtered and unfiltered car rides.

	Mean±SD	
	Filtered Ride(n = 40)	Unfiltered Ride(n = 40)
**Particulate matter<2.5μm(μg/m** ^**3**^ **)CabinPAPR**		
	15.4±4.9	15.0±5.4
	1.68±0.88^a^	10.4±5.64
**Carbon monoxide**	1.06±0.64	1.06±0.63
**Nitrogen dioxide (ppb)**	15.3±8.3	13.9±6.9
**In vehicle temperature (°C)**	23.8±2.4	24.1±3.0
**Condensation particle count(#/cm** ^**3**^ **)CabinPAPR**		
	4.3x10^4^±1.6x10^4^	4.7x10^5^±2.2x10^5^
	49.8±83.7^b^	4.2x10^4^±2.0x10^4^
**Black carbon(μg/m** ^**3**^ **)**	6.3±3.7	5.8±3.6
**Relative humidity(%)**	25.9±10.4	26.2±9.93

The mean of all rides and standard deviations (SD) are shown. Temperature and CO were monitored with a Langan T15v monitor, while relative humidity was measured with a HOBO 8 Pro Series monitor. Nitrogen dioxide was collected on calibrated triethanolamine-coated Sep-Pak cartridges with an SKC model 224-XR air pump and analyzed by high pressure liquid chromatography. Particulate matter concentrations (TSI SidePak model AM510 aerosol monitor) and condensation particle counts (condensation particle counter (CPC) TSI model 3007) were collected at 1-minute intervals, both within the vehicle cabin and within the PAPR. The levels were significantly lower in the PAPR during filtered rides due to HEPA filtration as indicated by ^a^ and ^b^.

^a^p≤0.0001 compared to unfiltered ride.

^b^p≤0.0001 compared to unfiltered ride.

### Car ride effect on resting vascular nitrite concentrations

**Fig 1:**
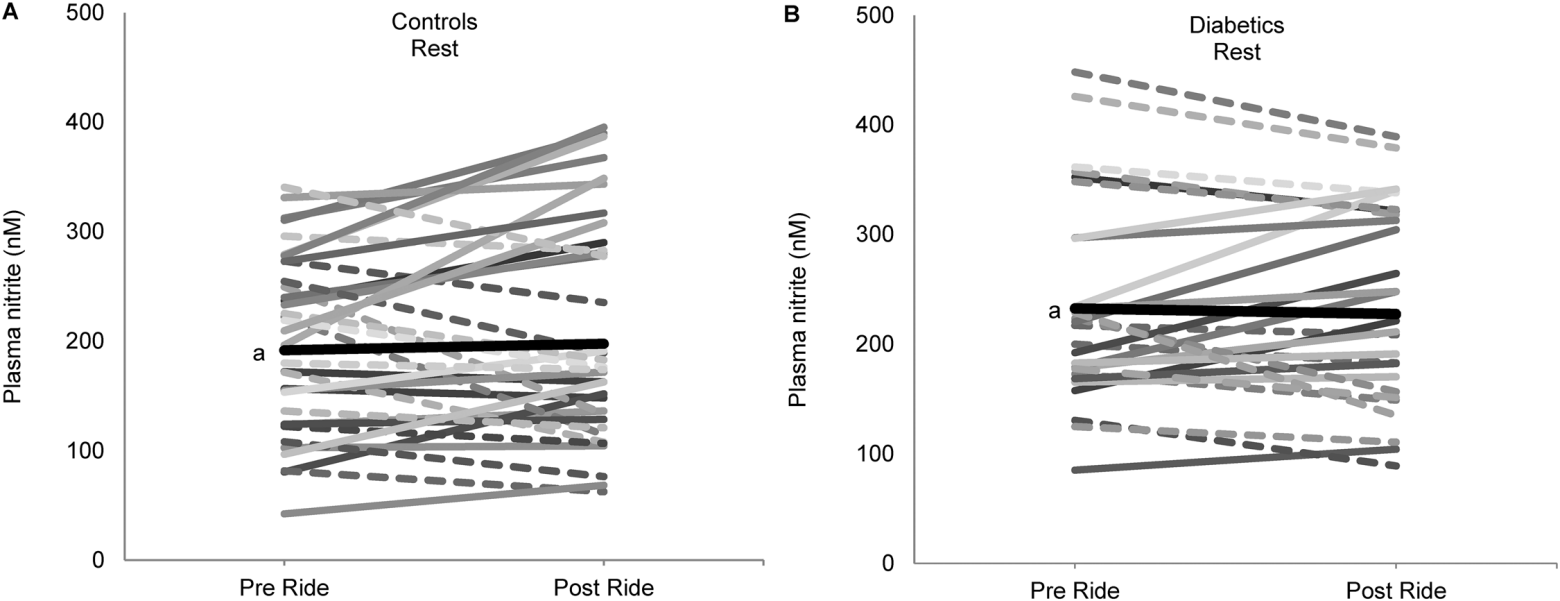
Acute ride (air pollution) effects on resting vascular nitrite levels (pre-ischemia). (A) Controls resting nitrite levels pre and post ride. (B) Diabetic resting nitrite levels pre and post ride. Dash lines indicate decreasing nitrite values, while solid lines indicate increasing values from pre to post ride. Unfiltered and filtered rides are combined. ^a^denotes mean values (thick black line).

We examined whether car ride pollutant exposures would reduce vascular NO immediately after a ride, either at rest or post-ischemia (hyperemia). The control group’s resting (pre-ischemia) plasma nitrite concentrations were found to be lower following unfiltered rides than filtered rides (18.3%, p = 0.058) (Fig 1A and Table 3). Diabetics showed no appreciable change from pre-ride values in pre-cuff nitrites following the ride (Fig 1B and Table 3).

**Table 3:** Effect of ischemic challenge and air pollution on vascular nitrite concentrations.

	Diabetes		Control		Ratio of Diabetes versus Control	
**Pre Car Ride** ^a^	***GeometricMean***	***95%CI(p-value)***	***GeometricMean***	***95%CI(p-value)***	***Ratio*** ^5^	***95%CI(p-value)*** ***
Rest(nM)^1^	230.1	191.2,276.8	182.6	155.6,214.1	1.26	0.99,1.61(p = .0623)***
	***Ratio***	***95%CI***	***Ratio***	***95%CI***	***Ratio***	***95%CI***
Reactivehyperemia^2^	1.02	0.89,1.17(p = .7991)*	1.16	1.03,1.31(p = .0170)*	0.87	0.73,1.06(p = .1584)***
**Car Ride Effect**	***Ratio***	***95%CI***	***Ratio***	***95%CI***	***Ratio***	***95%CI***
Rest^3^	0.97	0.79,1.20(p = .7654)**	0.82	0.66,1.01(p = .0580)**	1.19	0.89,1.60(p = .2342)***
Reactivehyperemia^4^	1.03	0.87,1.22(p = .6800)	1.05	0.90,1.23(p = .5041)	0.99	0.81,1.22(p = .9398) ***

^1^Pre-ischemia (Rest) nitrite levels were higher in diabetics (n = 15) than control subjects(230.1 vs. 186.2nM).

^2^The reactive hyperemic response caused a statistically significant increase in plasma nitrite levels in controls before the car ride (n = 20) but not those with diabetes. The car ride (exposure) effect is represented by the ratio of geometric means/ratios of the outcomes post-car ride from unfiltered to filtered rides, adjusting for pre-exposure levels.

^3^Diabetes status (diabetics (n = 15) vs controls (n = 20)) does not modify effects of exposure (unfiltered vs filtered rides).

^4^Furthermore, diabetes status did not modify the effects of exposure on vascular nitrite response during hyperemia (controls (n = 19) and diabetics (n = 15)).

^5^In the last 2 columns ratios of the measures for diabetes versus controls along with percent Confidence Intervals (CI) and p-values are presented. A value of one indicates no difference between people with different disease status

^a^ When examining the exposure effect, baseline values are included as covariates in the linear models rather than using raw differences between post- and pre-exposure levels as the outcome. By including the baseline value as a covariate, we allow for regression to the mean. This maintains reliability of the results in the case that there may be exposures en route to the session that influences the resting levels.

*p-value testing impact of reactive hyperemia on plasma nitrite levels

**p-value testing effect of exposure on either plasma nitrite levels or impact of reactive hyperemia

***p-value testing differences between diabetes and control.

### Vascular nitrite response to ischemic challenge

**Fig 2:**
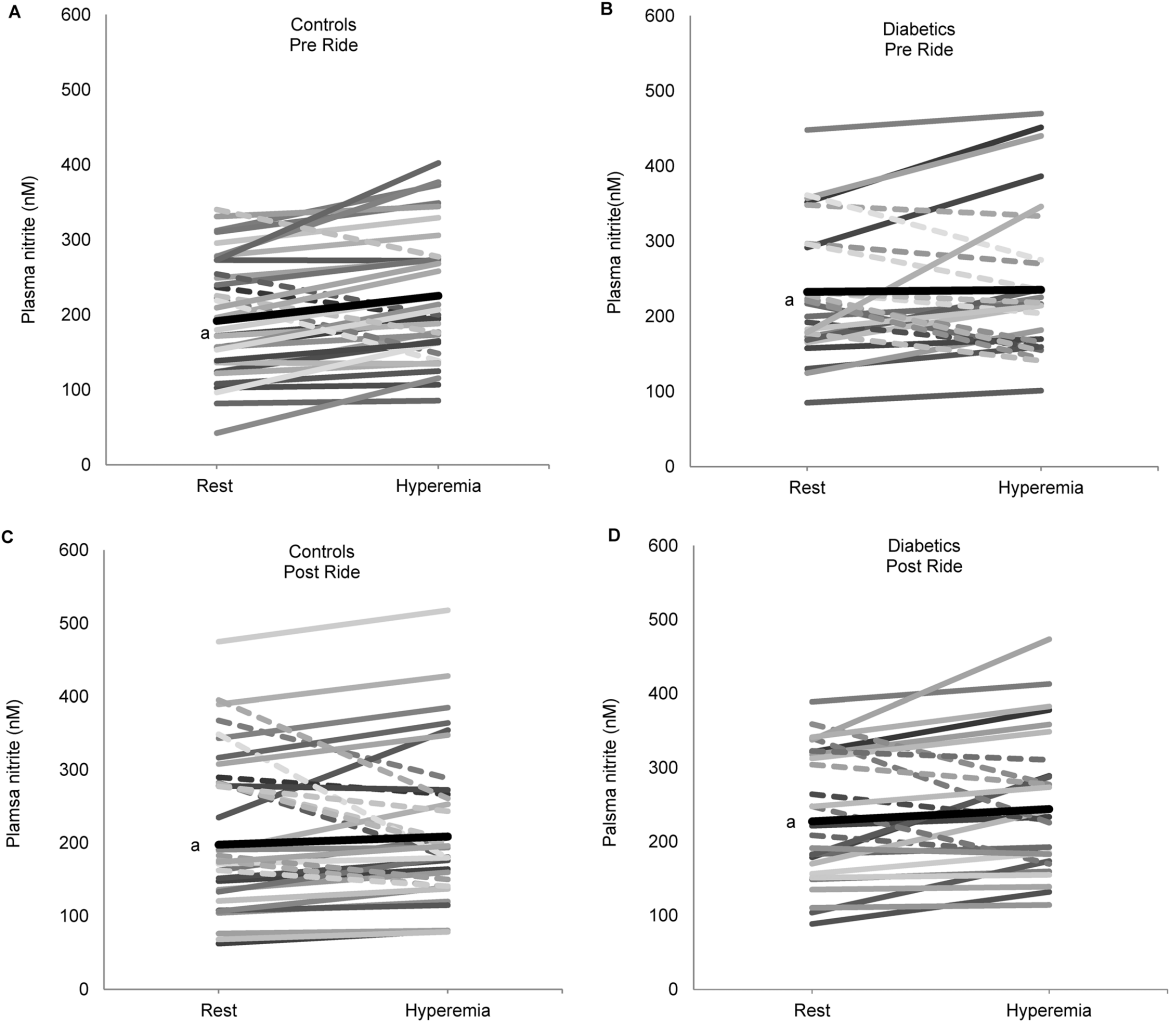
Vascular nitrite concentrations at rest and following ischemia in control and diabetic subjects. Blood draws were taken at rest and 60sec following release (hyperemia) of the five minute upper arm occlusion (50mmHg above SBP) before and following the ride. Dash lines indicate decreasing nitrite values, while solid lines indicate increasing values. Unfiltered and filtered rides are combined (A)Nitrite concentration (nM) changes in control subjects before the ride(n = 40); (B) Nitrite concentration (nM) changes in diabetic subjects before the ride(n = 30); (C) Nitrite concentration (nM) changes in control subjects following the ride(n = 30); (D) Nitrite concentration (nM) changes in diabetic subjects following the ride(n = 25). ^a^denotes mean values (thick black line).

Prior to the ride there was greater variability in resting nitrite levels within the diabetic group, as well as in their response to ischemic challenge, when compared to controls (Fig 2A vs. 2B). Geometric mean resting plasma nitrite levels were 26% higher in diabetic subjects than control subjects (Table 3).

Consistent with a healthy vascular response, controls showed a statistically significant 15.9% increase in plasma nitrite levels following ischemia (p = 0.017) prior to the car rides (Fig 2A and Table 3). In contrast, consistent with chronic endothelial dysfunction, nitrite concentrations following ischemia within the diabetic group did not show a significant increase (Fig 2B and Table 3).

### Car ride effect on vascular response to ischemic challenge

**Fig 3:**
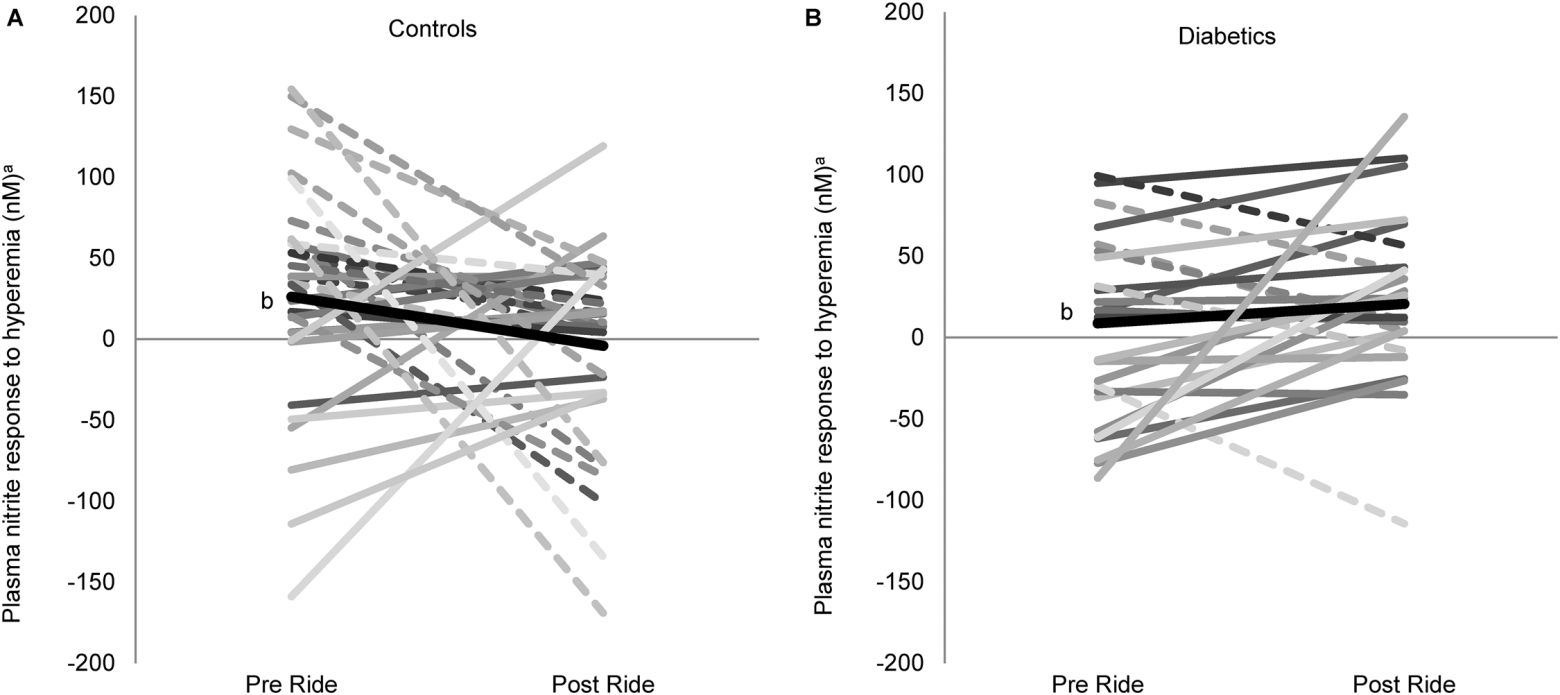
Air pollution exposure effects on vascular nitrite response to reactive hyperemia in controls and diabetics. A) Control subjects’ change in plasma nitrite response to ischemia (resting nitrites subtracted from reactive hyperemia nitrites)before (pre ride) and following (post ride) exposure (n = 34). Mean changes in nitrite values (pre ride: 26.1nM; post ride:-4.1nM). B) Diabetic subjects’ change in plasma nitrite response to ischemia before and following exposure (n = 25). Mean change in nitrite values (pre ride: 8.7nM; post ride: 20.6nM) Dash lines indicate decreasing nitrite values, while solid lines indicate increasing values. Unfiltered and filtered rides are combined. ^**a**^denotes (pre ischemia (resting) nitrite level) subtracted from (post ischemia (reactive hyperemia)nitrite level). ^**b**^denotes mean values (thick black line).

Following the car rides the significant nitrite increase from ischemia seen in controls prior to rides (15.9%, p = 0.017)(Fig 1A and Table 3) is no longer elicited in the unfiltered nor filtered condition, indicating a relative decrease in post-ischemia nitrite (and NO production) associated with the ride(5.0%, ns)(Fig 3A, Table 3, S1A and S1B Fig). There were no significant differences between filtered and unfiltered conditions within the diabetic group as they showed no significant change in response to ischemia following the ride (Fig 3B, S1C and S1D Fig).

## Discussion

This study set out to determine the impact of both type II diabetes and an acute exposure to TRAP on vascular reactivity through nitric oxide and its proximal metabolite, plasma nitrite. The findings of this study included (i) higher resting nitrite levels in diabetics than controls; (ii) a borderline significant decrease among control subjects in resting nitrite following the unfiltered car ride as compared to resting nitrites after the filtered ride; (iii) increased nitrites in controls but not diabetics during reactive hyperemia prior to exposure and abolition of this increase following acute TRAP exposure.

### Resting plasma nitrite levels

Our findings showed baseline nitrite levels in diabetics to be substantially elevated compared to controls. This is consistent with a previous study that showed subjects with endothelial dysfunction to have increased baseline vascular nitrites [45]. This increase could indicate that diabetics may tonically produce more NO to compensate for varying levels of vascular dysfunction (i.e. damaged endothelial cells) or reduced bioavailability of NO due to accelerated consumption by increased levels of circulating ROS.

Subjects with diabetes showed a small decrease in baseline nitrite levels following the unfiltered rides as compared to filtered rides, while controls displayed a more substantial, borderline significant, decrease. It is possible that TRAP may have a greater impact on the baseline level of NO in healthy vascular systems than on those with disease-related endothelial dysfunction and consequently a pre-existing increase in NO production.

### Plasma nitrite response to reactive hyperemia

Following reactive hyperemia but prior to the car ride, control subjects showed a significant increase in nitrite levels, while this response was blunted in diabetics. Allen showed a similar insufficient response in T2DM subjects following a graded exercise test (global reactive hyperemia inducer). It was anticipated that diabetics would have this limited ability to respond effectively to stimuli, as their vascular systems may be compromised by chronically higher levels of oxidative stress and elevated NO scavenging. Additionally, a study by O’Neill showed diabetic subjects to have lower flow-mediated vascular reactivity than those subjects considered at risk for diabetes. This physiological response is consistent with the lack of nitrite response to reactive hyperemic stimulation among diabetics in our study. As nitrite represents a proximal measure of eNOS function these results are consistent with reduced eNOS function as the molecular mechanism of this physiological response.

### Air pollution impact on reactive hyperemia

Our diabetic subjects displayed no change from their blunted pre-exposure reactive hyperemia response following the car ride. Their decreased responses during reactive hyperemia prior to exposure may indicate an already compromised vascular system, as discussed above. A recent study by Zanobetti (2014) showed no association between decreases in BAFMD and increases in ambient particulate pollution using a 5 day integrated sample in type II diabetics [46]. These data suggest that acute pollution exposure may not have a measureable impact on vascular response in those with pre-existing endothelial dysfunction.

The significant increase in plasma nitrite within the control group following reactive hyperemia is no longer present after exposure to TRAP (neither filtered nor unfiltered). This biochemical response is consistent with the observations of a study by Brook who found significant brachial artery vasoconstriction in a healthy population following acute exposure to a mixture of concentrated ambient fine particles and ozone [41]. The absence of an effect of particle filtration was not consistent with our recent studies of exhaled breath condensate nitrite [23] but is not inconsistent with human and animal studies of acute and chronic TRAP effects on vascular changes, where PM alone was not responsible for observed effects [47, 48]. These vascular responses are also in line with other previous animal studies, including a study by Ikeda (1995) that displayed increased vasoconstriction and inhibited dilation in rat aorta when exposed to diesel exhaust particles(DEP) or motorcycle exhaust [49]. The DEP response has been found to be related to NO-mediated dilators such as EDNO and NO donor drugs. These reactions may be attenuated by treatment with antioxidants such as SOD, thereby connecting the response with generation of ROS [50]. PM exposures done on healthy animals, hypertensive and atherosclerotic models have shown similar results. Single bolus instillations and prolonged inhalation studies have shown that these responses persist from several hours up to 24 hours. DEP and urban PM have been found to significantly raise blood pressure in animal models of hypertension (induced and genetic) and atherosclerosis [51–56]

### Location and duration of pollution exposure

In contrast to our findings, the diabetic subjects of the O’Neill study had altered vascular reactivity in association with multiple day increases in ambient air pollution, while the at-risk group displayed no change in response to air pollution. Since our diabetic subjects appeared to have severely comprised vascular NO responses prior to exposure, it is plausible that the impact of a relatively brief 2 hour car ride may not have been potent enough to further alter NO production in non-responsive systems. There is also a distinct possibility that physiological changes in brachial artery diameter occur even in the absence of significantly altered NO levels (i.e. due to nervous system alterations [57–59]).

The nature and length of exposure time may also play an important role in eliciting vascular changes. Our exposures were limited to 2 hours on highly trafficked roadways, while the O’Neill exposure metrics were average ambient urban pollution over 1–6 days in the Boston area. It is possible that a longer traffic exposure, repeated exposures over several days, or a later follow-up time may be necessary for impact on vascular reactivity. In addition, O’Neill also discussed sensitivity of diabetic subjects to pollution effects on vascular function as a possible reason for their findings; however, using the biochemical measure of nitrite, we found that diabetics appeared to be less sensitive than their non-diabetic counterparts to exposure to TRAP.

### Health status impact of control group

Our control population responded with more modest nitrite increases during reactive hyperemia than those previously reported. This may be due to their more advanced age, elevated BMIs, and co-morbid conditions (other than diabetes) compared with subjects in previous reports [45]. The control subjects also had a significantly greater number of women than the diabetic group, which could account for a sex-related variation between the 2 groups. Additionally, medication differences between the two groups may alter vascular reactivity and NO production. For instance, consistent with their cardiovascular risk profile, 90% of the diabetic subjects were taking statins, in comparison to 20% of the controls. The marginal increase in diabetic resting plasma nitrite compared to controls could potentially be due to antioxidant effects of statins, in addition to potential compensatory mechanisms [9, 12, 60].

### Conclusion

This study demonstrated the adverse vascular effects of a two hour traffic exposure in an older non-diabetic population. The most pronounced change was seen in the decreased vascular reactivity response in non-diabetics following a car ride associated with increased TRAP inhalation. Before exposure, type II diabetic subjects demonstrated a decreased ability to respond appropriately to a vascular stressor and this was not altered by the TRAP exposure. Since the diabetic subjects show pre-existing compromise of their vascular systems, the extent to which a TRAP exposure affected them is unknown. The decrease of resting nitrite levels after TRAP pollution inhalation, and lowered vascular reactivity in non-diabetics, supports the proposition that real-world TRAP acutely increases endothelial dysfunction and alters NO metabolism leading to inadequate vascular responses to hypoxia. These results further validate the previously observed detrimental effects of TRAP exposure on vascular reactivity in controlled diesel exposure studies, and provides a direct mechanism for the epidemiological observations of MI associated with acute traffic exposure [43, 61–63].

## Supporting Information

S1 Fig Air pollution exposure effects due to filtered and unfiltered ride on vascular nitrite response to reactive hyperemia in controls and diabetics. (A)Control subjects’ change in plasma nitrite response to ischemia (resting nitrites subtracted from reactive hyperemia nitrites)before (pre ride) and following (post ride) exposure to filtered ride (n = 16). Mean changes in nitrite values (pre ride: 14.9nM; post ride: -2.9nM). (B) Control subjects’ change in plasma nitrite response to ischemia before and following exposure to unfiltered ride (n = 17). Mean changes in nitrite values (pre ride: 36.7nM; post ride: 3.8nM). (C)Diabetic subjects’ change in plasma nitrite response to ischemia before and following filtered ride (n = 14). Mean change in nitrite values (pre ride: 13.0nM; post ride: 30.3nM) (D)Diabetic subjects’ change in plasma nitrite response to ischemia before and following unfiltered ride (n = 13). Mean change in nitrite values (pre ride: 20.8nM; post ride: 43.7nM). ^**a**^denotes (pre ischemia (resting) nitrite level) subtracted from (post ischemia (reactive hyperemia)nitrite level). ^**b**^denotes mean values (thick black line).
(TIF)
