# Supplementary figures and images for: Disrupted Nitric Oxide Metabolism from Type II Diabetes and Acute Exposure to Particulate Air Pollution
Source: PLoS One. 2015 Dec 14;10(12):e0144250. doi: 10.1371/journal.pone.0144250 (PMC4682772; doi:10.1371/journal.pone.0144250)

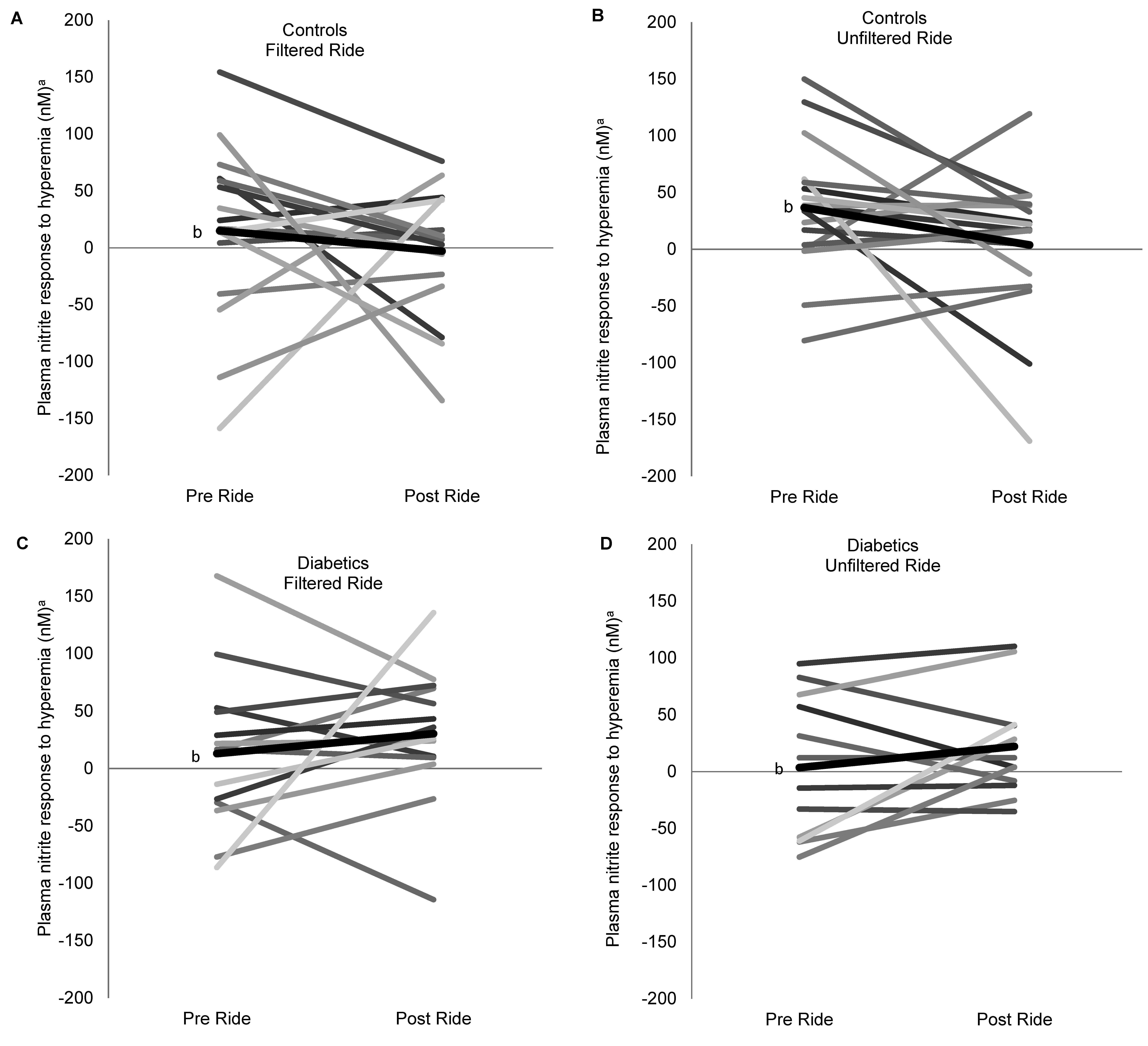

Supplement: S1 Fig — (A)Control subjects’ change in plasma nitrite response to ischemia (resting nitrites subtracted from reactive hyperemia nitrites)before (pre ride) and following (post ride) exposure to filtered ride (n = 16). Mean changes in nitrite values (pre ride: 14.9nM; post ride: -2.9nM). (B) Control subjects’ change in plasma nitrite response to ischemia before and following exposure to unfiltered ride (n = 17). Mean changes in nitrite values (pre ride: 36.7nM; post ride: 3.8nM). (C)Diabetic subjects’ change in plasma nitrite response to ischemia before and following filtered ride (n = 14). Mean change in nitrite values (pre ride: 13.0nM; post ride: 30.3nM) (D)Diabetic subjects’ change in plasma nitrite response to ischemia before and following unfiltered ride (n = 13). Mean change in nitrite values (pre ride: 20.8nM; post ride: 43.7nM). adenotes (pre ischemia (resting) nitrite level) subtracted from (post ischemia (reactive hyperemia)nitrite level). bdenotes mean values (thick black line). (TIF) [file pone.0144250.s001.tif]
